# Survival Rate and Short-Term Outcomes of Periviable Preterm Infants: A Single-Center Experience From the United Arab Emirates

**DOI:** 10.7759/cureus.73877

**Published:** 2024-11-17

**Authors:** Mustafa Alabdullatif, Fahad Butt, Aimen BenAyad, Aiman Rahmani

**Affiliations:** 1 Neonatology, Tawam Hospital, Al Ain, ARE; 2 Pediatrics and Neonatology, Tawam Hospital, Al Ain, ARE

**Keywords:** extreme preterm, limit of viability, periviable birth, short-term outcome, survival rate

## Abstract

The limit of periviability is constantly changing as infants born at 22-25 weeks of gestation increasingly survive. The data from our region are limited due to the small numbers of these infants among the NICU population.

In this study, we evaluated the survival rates and short-term outcomes among preterm neonates between 22 and 24 weeks of gestation admitted to Tawam Hospital, United Arab Emirates.

Our retrospective analysis included 100 cohorts of newborns from 22 to 25 weeks of gestation throughout the eight-year period in our level 3 NICU. We evaluated the use and effects of prenatal steroids and intrapartum antibiotics, in addition to perinatal complications, and examined their outcomes (survival, length of stay, and major morbidities).

The survival rate of our periviable neonates was 18% (N = 18/100). Only one 22-week infant out of 32 cases (3%) survived during the study period in 2023. In contrast, the survival rate of infants of 23- and 24-week gestational age was 10% (N = 4/40) and 46% (N = 13/28), respectively.

Although our focus in this study was to evaluate the survival of neonates who were born at around the limits of viability, we also reported the short-term outcomes at our single center, including intraventricular hemorrhage (IVH), periventricular leukomalacia (PVL), bronchopulmonary dysplasia (BPD), retinopathy of prematurity (ROP), and necrotizing enterocolitis (NEC).

Our data demonstrate that the trend of increasing survival with higher gestational age continues to improve over time. However, there was a significant risk of short-term co-morbidities for those who survived. Further studies are required to have robust data on short and long-term outcomes for this population.

The information provided by this study could be essential for counseling parents, enabling them to participate actively in formulating their infants' care plans. It may also help parents and healthcare professionals reach a more informed and collaborative decision regarding active resuscitation and subsequent care plans for these periviable neonates.

## Introduction

Throughout history, advancements in neonatal care have continuously advanced the viability limit. The limit of viability is defined as the minimum age at which a fetus can survive outside of the uterus [1]. It is sometimes defined as a certain gestational age or birthweight at which over 50% of infants survive to discharge [2,3]. There is no definitive cut-off for gestational age or weight at which a human fetus is automatically considered viable [1]. The limit of viability needs to be better established; a universal definition may not be feasible due to practice variations among individuals, medical facilities, and countries [4]. The neonatologist usually considers the age of viability generally to be around 24 weeks of gestation [5]. Periviable births usually refer to neonates born between 22 0/7 and 24 6/7 weeks of gestation [6]. For periviable preterm infants, the data are still limited about the outcome of this new population.

Internationally, survival rates for infants born at 22 to 24 weeks of gestation vary widely. For instance, neonatal survival rates vary from one country to another depending on multiple factors. In the past decade, survival rates to discharge for newborns delivered at 23-24 weeks of gestation in the United States, England, and Australia were approximately 23-27% at 23 weeks and 42-59% at 24 weeks of gestation [4-17]. For 22 weeks, the survival rates are around 0.7% in France to 2.0% in the United Kingdom, 5.1% in the United States, 9.8% in Sweden, and 33.1% in Japan [7].

Evaluating the quality of survival is crucial. Literature on neurodevelopmental outcomes indicates that infants at ≤ 25 gestational weeks (GW) survive without or with minimal impairments in 6-20%. In contrast, less than 5% of those infants born at 22 and 23 weeks do so [15]. Studies from developed countries like the United States, Sweden, and the United Kingdom showed similar data regarding mild, moderate, and severe disabilities, which correlate with gestational age. As gestational age decreases, the incidence of disabilities increases [11,14,16].

Parents and healthcare professionals collaborate to make decisions regarding active resuscitation and subsequent care plans for those periviable neonates. Gestational age is the primary factor. Other factors, such as birth weight, gender, multiple gestations, and exposure to antenatal steroids, may also influence outcomes [17,18]. Providing realistic survival and outcome data is essential for counseling parents, enabling them to participate actively in formulating care plans. While such data are crucial for parent counseling, it must be emphasized that these are statistical probabilities, not specific predictions for their situation [19].

## Materials and methods

Objectives

To evaluate the survival rates and short-term outcomes (intraventricular hemorrhage (IVH), periventricular leukomalacia (PVL), bronchopulmonary dysplasia (BPD), retinopathy of prematurity (ROP), and necrotizing enterocolitis (NEC)) of preterm neonates between 22 and 24 weeks of gestation admitted to Tawam Hospital, United Arab Emirates, over seven years from January 2016 to June 2023. Tawam Hospital ranks among the largest tertiary teaching hospitals in the SEHA corporate - United Arab Emirates (UAE), handling approximately 3,000 deliveries annually and housing a level 3+ NICU with a capacity of 51 beds.

Definitions

Neonates were considered to have received active treatment if they received any of the following interventions in the delivery room: ventilatory support (invasive or non-invasive), tracheal intubation, chest compressions, or epinephrine. Gestational age (GA) was determined by the attending obstetrician and recorded in the mother’s file. Small for GA (SGA) was defined as birth weight <10th percentile for age, according to the Fenton growth chart [20]. While large for GA (LGA) was defined as birth weight >90th percentile for age, according to the Fenton growth chart [20]. Antenatal steroid treatment was considered completed if two doses were given 24 hours before delivery and partial if any dose was given less than 24 hours before delivery. Survivors were considered those who survived to the time of discharge. IVH was diagnosed based on cranial ultrasound scanning during the NICU stay, classified using Papile’s scale [21]. Severe IVH was defined as grade 3 or 4 of IVH according to Papile’s scale. PVL was defined as periventricular echogenicity on a cranial ultrasound scan. ROP was defined using the standard international classification [22]. ROP is considered severe if the unilateral or bilateral disease requires retinal therapy in at least one eye. BPD was defined as the need for supplemental oxygen at 36 weeks corrected age [23]. Patent ductus arteriosus (PDA) was considered significant when medical or surgical treatment was required. NEC was diagnosed according to the modified Bell's criteria of stage two or higher [24].

Methods

A retrospective cohort study was conducted between January 2016 and June 2023. The study included periviable neonates admitted to Level III NICUs at Tawam Hospital, Al Ain, Abu Dhabi, who met the inclusion criteria (gestational age between 22 and 24 weeks and received active resuscitation). Demographic data, perinatal complications, various interventions, and outcomes (survival, length of stay, and major morbidities) were collected from electronic medical records.

Inclusion criteria

Our retrospective study included all infants with a gestational age of 22 weeks 0 days to 24 weeks 6 days born at Tawam Hospital from January 2016 to June 2023.

Exclusion criteria

Neonates were excluded if they had any major congenital anomalies, significant chromosomal abnormalities, stillbirth, or did not receive active resuscitation.

Data collection

A data sheet was developed to gather information from patients' electronic medical records using Cerner EMR software (Oracle Health, Kansas City, MO). This information covered gestational age in weeks and days, birth weight in grams, gender, antenatal treatments (including steroids, magnesium sulfate, and antibiotics), chorioamnionitis, plurality, maternal age, mode of delivery, and whether active treatment was provided to each included infant. Additionally, the data sheet recorded outcomes such as survival until discharge from the NICU, grades of intracranial hemorrhage (IVH), periventricular leukomalacia (PVL), bronchopulmonary dysplasia (BPD), retinopathy of prematurity (ROP), and necrotizing enterocolitis (NEC). For infants who did not survive, details such as age at death, whether the death occurred in labor, delivery, or in the NICU, and the direct cause of death were documented.

Ethical approval

This study was approved by the SEHA Research Ethics Committee (reference number: HREC SEHA-IRB-449) and the Abu Dhabi Health Research and Technology Ethics Committee (reference number: DOH/CVDC/2023/1193).

Statistical analysis

Statistical analysis was performed using SPSS version 28 (IBM Corp., Armonk, NY). Continuous variables were summarized as mean ± standard deviation (SD), while categorical variables were reported as numbers (percentages). Chi-square tests and Fisher’s exact tests were used to evaluate the association between categorical variables. A p-value < 0.05 was considered statistically significant.

## Results

Study population

**Figure 1:**
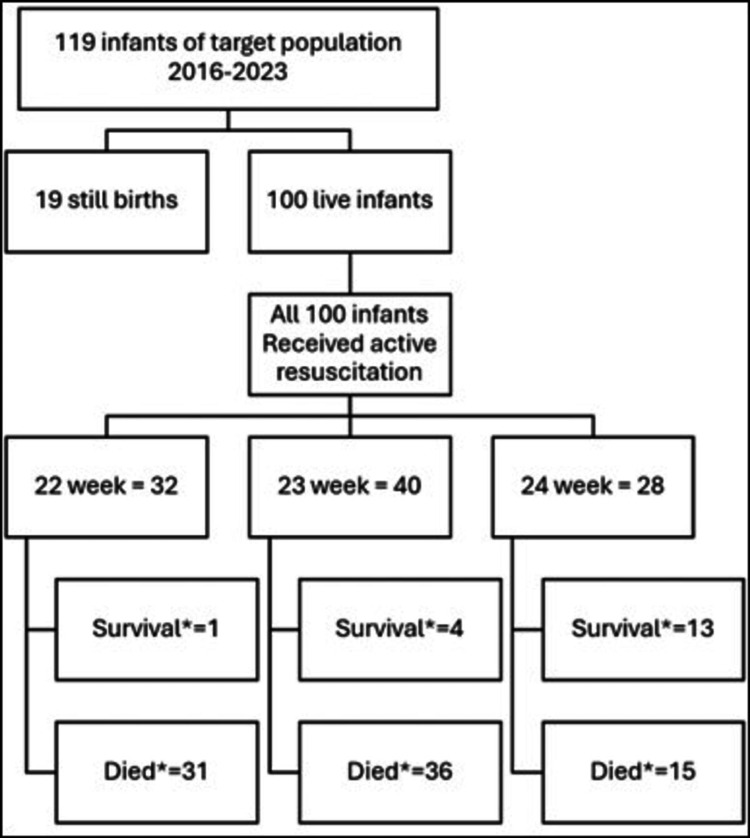
Flowchart the study. * Number of cases.

Our study included 119 infants, of whom 19 were stillbirths, and the remaining 100 infants, between gestational ages 22+0 and 24+6, met the inclusion criteria. The mean (SD) birth weight for gestational ages 22, 23, and 24 weeks was 522 g (88), 557 g (73), and 646 g (114), respectively (Figure 1).

A total of 32 infants were delivered at 22 weeks, with a mean (SD) weight of 522 (88) grams. A total of 40 infants were delivered at 23 weeks, with a mean (SD) weight of 557 (74) grams. In contrast, 28 cases were delivered at 24 weeks, with a mean (SD) weight of 646 (114) grams. Of the study population, 69% (N = 69/100) were males, and 31% (N = 312/100) were females. A total of 78% (N = 78/100) of these infants were born to Emirati families, while 22% (22/1000) were expats. The mean (SD) age of mothers across the study population was 32 (6). A total of 30% (N = 30/100) of infants were conceived by in vitro fertilization (IVF) and 56% (N = 56/100) of the deliveries were multiple births. All the babies were in-born. Spontaneous vaginal delivery was the most common mode of delivery at 77% (N = 77/100) (Table 1)*.*

**Table 1:** The basic characteristic of the study’s population. N: number of cases; SD: standard deviation.

Demographic	22 weeks (N = 32)	23 weeks (N = 40)	24 weeks (N = 28)	Whole study population (N = 100)	P-value
Birth weight (grams), mean (SD)	522 (88)	557 (74)	646 (114)	571 (103)	<0.001
Mother’s age, mean (SD)	33 (6)	30 (6)	34 (5)	32 (6)	0.049
Gender					
Male, N (%)	25 (78)	27 (68)	17 (61)	69 (69)	0.339
Female, N (%)	7 (22)	13 (32)	11 (39)	31 (31)	
Nationality					
Emirati, N (%)	20 (63)	36 (90)	22 (79)	78 (78)	0.020
Expats, N (%)	12 (37)	4 (10)	6 (21)	22 (22)	
Mode of conception					
Spontaneous, N (%)	19 (59)	30 (75)	21 (75)	70 (70)	0.282
In vitro fertilization (IVF), N (%)	13 (41)	10 (25)	7 (25)	30 (30)	
Multiple births					
Singleton, N (%)	14 (44)	17 (43)	13 (46)	44 (44)	0.008
Twin, N (%)	9 (28)	20 (50)	15 (54)	44 (44)	
Triplet, N (%)	9 (28)	3 (7)	0 (0)	12 (12)	
Delivery location					
Inborn, N (%)	32 (100)	40 (100)	28 (100)	100 (100)	-
Outborn, N (%)	0 (0)	0 (0)	0 (0)	0 (0)	
Delivery mode					
Vaginal delivery, N (%)	29 (91)	31 (77)	17 (61)	77 (77)	0.023
Cesarean section, N (%)	3 (9)	9 (23)	11 (39)	23 (23)	

Perinatal complications and interventions

The incidence of premature rupture of membrane (PROM) and chorioamnionitis in our study population was 20% (N = 20/100) and 10% (N = 10/100), respectively (Table 2).

**Table 2:** Demographic of perinatal care. N: number of cases; SD: standard deviation; * P-value < 0.05 was considered statistically significant.

Demographic	22 weeks (N = 32)	23 weeks (N = 40)	24 weeks (N = 28)	Whole study population (N = 100)	P-value*
Premature rupture of membranes (PROM), N (%)	8 (25)	11 (28)	1 (4)	20 (20)	0.042
Chorioamnionitis, N (%)	4 (13)	4 (10)	2 (7)	10 (10)	0.811
Antenatal steroids ≥ 1 dose, N (%)	14 (44)	27 (68)	22 (79)	63 (63)	0.001*
Antenatal magnesium sulfate given, N (%)	0 (0)	12 (30)	4 (14)	16 (16)	0.003*
Intrapartum antibiotics given, N (%)	11 (34)	20 (50)	9 (32)	40 (40)	0.246
Apgar at 1 min, mean (SD)	4 (2)	4 (2)	5 (1)	4 (2)	0.114
Apgar at 5 min, mean (SD)	5 (2)	6 (2)	7 (2)	6 (2)	
Apgar at 10 min, mean (SD)	6 (2)	7 (2)	7 (1)	7 (2)	
Intubation at resuscitation, N (%)	32 (100)	40 (100)	28 (100)	100(100)	-
Required umbilical vein catheter insertion at resuscitation, N (%)	1 (3)	2 (5)	1 (4)	4 (4)	0.913
Normal saline bolus at resuscitation, N (%)	1 (3)	1 (3)	1 (4)	3 (3)	0.967
Required chest compression or epinephrine during resuscitation, N (%)	4 (13)	4 (10)	2 (7)	10 (10)	0.788
Admitted to NICU, N (%)	31 (97)	39 (98)	27 (96)	97 (97)	0.967

Peripartum care practices

Perinatal interventions such as perinatal antibiotics, antenatal steroids (at least one dose), and antenatal magnesium sulfate in our population were required by 40% (N = 40/100), 62% (N = 63/100), and 16% (N = 16/100), respectively. Antenatal steroids were given (at least one dose) to 44%, 68%, and 79% (P = 0.001) of 22-, 23-, and 24-week infants, respectively (P = 0.001). Magnesium sulfate before delivery was given to 0%, 30%, and 14% for 22, 23, and 24 weeks, respectively (P = 0.003). Intrapartum antibiotics were given to 34%, 50%, and 32% of 22-, 23-, and 24-week infants, respectively (P = 0.246) (Table 2)*.*

Neonatal resuscitation in the delivery room

All our neonates received active resuscitation in the delivery room, including endotracheal intubation. The Apgar score was 4, 6, and 7 at one, five, and 10 minutes, respectively (P = 0.114).

As demonstrated in Table 2, intensive resuscitation (cardiopulmonary resuscitation with or without epinephrine) was required in 13% (N = 4/32), 10% (N = 4/40), and 7% (N = 2/28) of cases for 22-, 23-, and 24-week neonates (P = 0.788). A total of 97 out of 100 neonates responded to resuscitation in the delivery room. However, three infants did not survive in the delivery room, with one case in each gestational age of 22, 23, and 24 weeks.

Short-term outcomes

Survival

**Figure 2:**
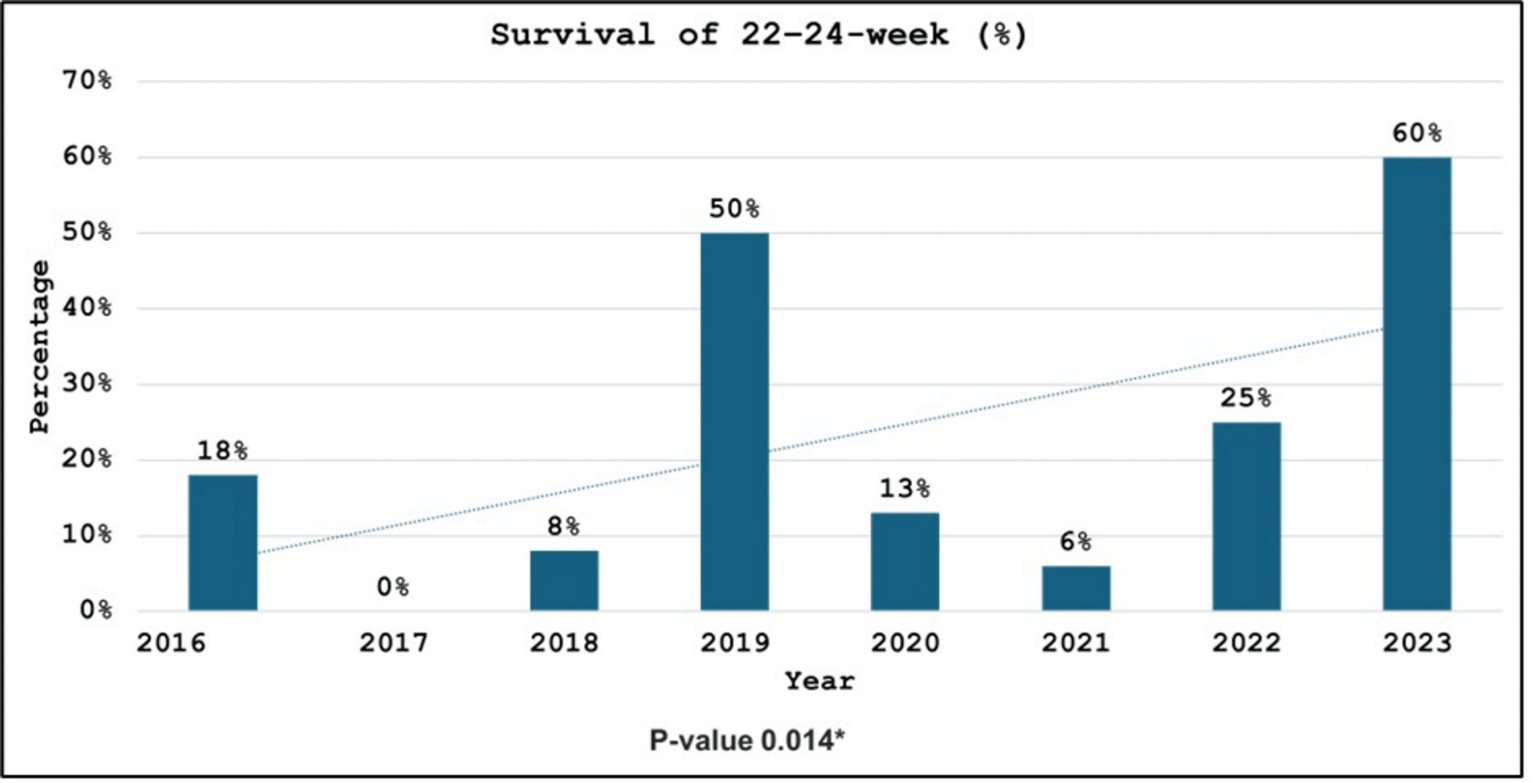
Survival of 22-, 23-, and 24-week gestational age group neonates in the study period from 2016 to 2023. * P-value < 0.05 was considered statistically significant.

**Figure 3:**
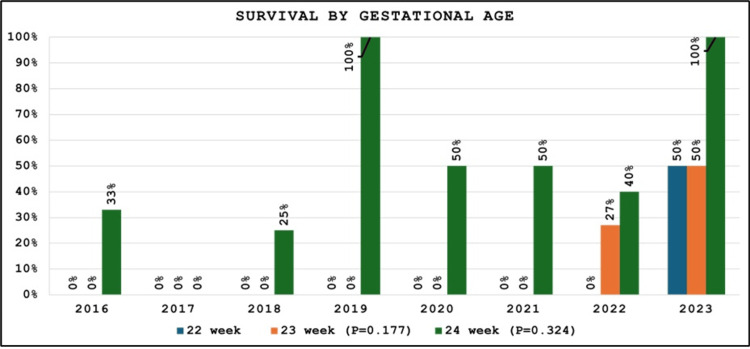
Survival of each gestational age elucidated separately over the study period from 2016 to 2023.

The survival rate of our periviable neonates was 18% (N = 18/100). Only one 22-week infant out of 32 cases (3%) survived during the study period in 2023. In contrast, the survival rate of infants of 23- and 24-week gestational age was 10% (N = 4/40) and 46% (N = 13/28), respectively. As shown in Figure 2, there was a statistically significant rise in the survival of preterm infants from the target population from 18% (N = 3/17) in 2016 to 60% (N = 3/5) in 2023 (P = 0.014). There was also a rise in survival across all gestations over time, but it did not reach statistical significance (Figure 3).

The length of stay of preterm babies who passed away was inversely related to gestation; the mean (SD) length of stay for 22, 23, and 24 weeks was 4 (7), 23 (42), and 34 (51) days, respectively.

Short-term outcomes for the surviving cases

Length of Stay

**Figure 4:**
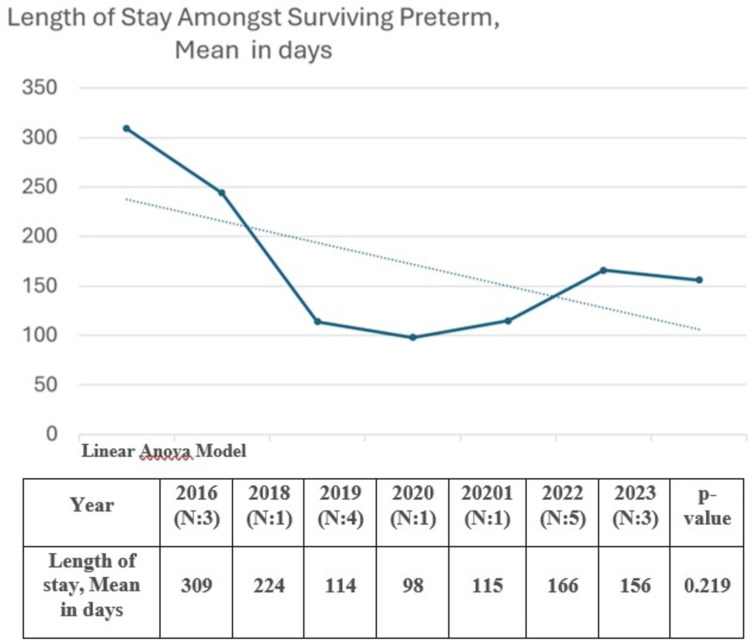
Length of stay of surviving neonates delivered at 23 and 24 weeks over the study period.

Length of stay was directly proportional to gestational age with Pearson correlation coefficient <0.0001. The length of stay and median (IQR) for the surviving preterm infants at 23- and 24-week gestation was 180 (166, 187) and 123 (111, 193) days, respectively, as demonstrated in Table 3. While the duration of stay for only the surviving case of 22-week gestation was 154 days. During our study period, the mean (SD) length of stay of surviving 23- and 24-week infants decreased over time from 309 (208) days in 2016 to 156 (24) days in 2023 (P = 0.219), as demonstrated in Figure 4.

**Table 3:** Short-term outcomes in 23- and 24-week survivors in the study period from 2016 to 2023. N: number of cases; IQR: interquartile range.

Outcomes	23 weeks (N = 4)	24 weeks (N = 13)
Length of stay (days), median (IQR)	180 (166, 187)	123 (111, 193)
Conventional ventilator, N (%)	4 (100)	13 (100)
High-frequency ventilation, N (%)	4 (100)	8 (62)
Required surfactant ≥ 1 dose, N (%)	4 (100)	13 (100)
Pneumothorax needing intervention, N (%)	0 (0)	2 (15)
Sepsis, N (%)	2 (50)	9 (69)
Meningitis, N (%)	0 (0)	2 (15)
Shock, N (%)	1 (25)	3 (23)
Acute kidney injury, N (%)	3 (75)	4 (31)
Intraventricular hemorrhage grade 3 or above, N (%)	1 (25)	3 (23)
Periventricular leukomalacia, N (%)	0 (0)	1 (8)
​​Necrotizing enterocolitis grade 2 or above, N (%)	2 (50)	4 (31)
Retinopathy of prematurity required treatment, N (%)	3 (75)	6 (46)
Bronchopulmonary dysplasia, N (%)	4 (100)	13 (100)

Short-term morbidities

Twenty-Two-Week Gestation

The sole surviving case of 22-week gestation had multiple co-morbidities. The baby developed severe bronchopulmonary dysplasia, culture-proven sepsis, acute kidney injury, and severe retinopathy of prematurity.

Twenty-Three & 24-Week Gestation

The short-term morbidities of 23- and 24-week gestation infants are demonstrated in Table 3.

Respiratory: High-frequency ventilation (HFOV) was needed in 100% (4/4) of cases for 23-week gestation, while 62% (N = 8/13) of cases for 24-week gestation were given HFOV. Pneumothorax needing intervention was observed in 15% (N = 2/13) of 24-week gestation cases, whether none of the infants of 23-week gestation had pneumothorax. All survivors in the 23- and 24-week age group developed bronchopulmonary dysplasia.

Necrotizing enterocolitis: A total of 50% (N = 2/4) of 23-week infants who survived developed NEC; both had surgical NEC. Four cases out of 13 (30%) of 24-week preterm infants developed NEC; three had medical NEC, and one developed surgical NEC.

Acute kidney injury (AKI): AKI was present in 75% (N = 3/4) of 23-week preterm infants and 30% (N = 4/13) of 24-week preterm infants.

Intraventricular hemorrhage (IVH) and periventricular leukomalacia (PVL): The incidence of severe IVH in the 23- and 24-week gestational age group was 25% (N = 1/4) and 23% (N = 3/13), respectively. There was one case with PVL in the 24-week group. No case of PVL was observed in 23 weeks.

Severe retinopathy of prematurity (ROP): The ROP was developed in 75% (N = 3/4) of the 23-week group, while 46% (N = 6/13) of the 24-week group had it.

PDA: Hemodynamic instability and shock were more common in the smaller gestational age group: 25% (N = 1/4) at 23 weeks and 23% (N = 3/13) at 24 weeks.

Infection: The 24-week neonates had a higher incidence of proven sepsis and meningitis, at 69% (N = 9/13) and 15% (N = 2/13), respectively, compared to 50% (N = 2/4) and 0% (0/4) in the 23-week neonates, respectively.

## Discussion

Our study showed that the survival rate of periviable preterm newborns is as low as 18%. This rate decreases dramatically from 46% at 24 weeks of gestation to 10% and 3% for 23 and 22 weeks, respectively. Similar results were demonstrated in many studies in our region, which showed a survival rate of 35% and 64% for 23- and 24-week gestation [25,26]. Our study showed some improvement in the survival rate of those periviable preterm. Contrary to other studies [26,27], there were no changes in the survival rate for 23- and 24-week gestation overtime.

Our findings showed there is a significant risk of short-term morbidities in our population of those who survived with the incidence of BPD (100%), severe IVH (23 %), PVL (6%), ROP (53%), sepsis (65%), and NEC (35%). These incidences are comparative to another study with larger numbers of infants, which demonstrated the following outcomes: BPD (58%), severe IVH (12%), PVL (4%), ROP (30%), sepsis (58%), and NEC (12%) [27].

Strengths and limitations

To our knowledge, our study is among the first to look at the short-term outcomes of newborns around the age of viability in our region. Nevertheless, our study's limitations include retrospective data collection from medical records, a small sample size reflecting a single-center experience, and evaluation of the short-term outcomes only with little attention to long-term or neurodevelopment outcomes. Therefore, our data should be interpreted with caution.

## Conclusions

The survival rate was 3%, 10%, and 46% for 22-, 23- and 24-week gestational age, respectively. This survival rate for periviable preterm babies is still low. However, it increases with higher gestational age and continues to improve over time. There is a significant risk of short-term co-morbidities in the survivors. The information provided by this study could be essential for counseling parents, enabling them to participate actively in formulating their infants' care plans. Further studies are required to have robust data on short- and long-term outcomes for this population.
